# Evaluation of an association between plasma total homocysteine and schizophrenia by a Mendelian randomization analysis

**DOI:** 10.1186/s12881-015-0197-7

**Published:** 2015-07-26

**Authors:** Shusuke Numata, Makoto Kinoshita, Atsushi Tajima, Akira Nishi, Issei Imoto, Tetsuro Ohmori

**Affiliations:** Department of Psychiatry, Institute of Biomedical Sciences, Tokushima University Graduate School, 3-18-15, Kuramoto, Tokushima 770-8503 Japan; Department of Bioinformatics and Genomics, Graduate School of Medical Sciences, Kanazawa University, 13-1, Takawamachi, Kanazawa, Ishikawa 920-8640 Japan; Department of Human Genetics, Institute of Biomedical Sciences, Tokushima University Graduate School, 3-18-15, Kuramoto, Tokushima 770-8503 Japan

## Abstract

**Background:**

The results of meta-analyses conducted by previous association studies between total homocysteine and schizophrenia suggest that an elevated total homocysteine level is a risk factor for schizophrenia. However, observational studies have potential limitations, such as confounding and reverse causation. In the present study, we evaluated a causal relationship between plasma total homocysteine and schizophrenia by conducting a Mendelian randomization analysis.

**Methods:**

We used the *MTHFR* C677T polymorphism as an instrumental variable, which affects the plasma total homocysteine levels. To calculate the risk estimate for the association of this single nucleotide polymorphism (SNP) with schizophrenia, we conducted a meta-analysis of case–control studies that comprise a total of 11,042 patients with schizophrenia and 14,557 control subjects. We obtained an estimate for the association of this SNP with the plasma total homocysteine levels from a meta-analysis of genome-wide association studies comprising 44,147 individuals.

**Results:**

By combining these two estimates, we demonstrated a significant effect of the plasma total homocysteine on schizophrenia risk, representing an OR of 2.15 (95 % CI = 1.39–3.32; p = 5.3 x 10^−4^) for schizophrenia per 1-SD increase in the natural log-transformed plasma total homocysteine levels.

**Conclusions:**

We provided evidence of a causal relationship between the plasma total homocysteine and schizophrenia, and this result will add insight into the pathology and treatment of schizophrenia.

**Electronic supplementary material:**

The online version of this article (doi:10.1186/s12881-015-0197-7) contains supplementary material, which is available to authorized users.

## Background

Homocysteine is a key substance in the methionine cycle, which is involved in one-carbon methyl group-transfer metabolism. The methylenetetrahydrofolate reductase (*MTHFR*) C677T (rs1801133) polymorphism is a well-characterized genetic variant. C677T of the *MTHFR* gene results in amino-acid substitution (Ala222Val), and causes a reduction of enzyme activity and higher plasma total homocysteine levels [1]. The association of this variant with the plasma total homocysteine was confirmed by a recent meta-analysis of genome-wide association studies [2]. Previous meta-analyses of association studies between total homocysteine, which includes plasma and serum total homocysteine, and schizophrenia suggest that an elevated total homocysteine level is a risk factor for schizophrenia [3, 4]. However, observational studies have potential limitations, such as confounding, reverse causation, and selection bias. In fact, several findings of observational studies have been shown to be spurious causes by subsequent randomized controlled trials, such as hormone replacement therapy in coronary heart disease, β carotene in lung cancer, and vitamin E and vitamin C in cardiovascular disease [5]. In addition to genetic variants [2], many determinants that affect plasma total homocysteine concentrations, including physiologic determinants, such as age and sex, lifestyle determinants, such as vitamin intake, smoking and coffee, and clinical conditions, such as folate deficiency and renal failure, have been reported [6]. Moreover, whether hyper-homocysteine itself causes schizophrenia or schizophrenia causes hyper-homocysteine has not been clarfied. For examples, the high prevalence of smoking and decreased folate levels in patients with schizophrenia have been reported [7–10], both of which are known to be associated with increased plasma total homocysteine concentrations.

Mendelian randomization analysis, which uses genetic variants as instrumental variables for exposures of interest, can overcome problems of confounding and reverse causality, and is a useful method for assessing causal relationships in epidemiological studies [11–16].

Mendelian randomization refers to the random allocation of alleles at the time of gamete formation. A specific genotype carried by a person results from two such randomized transmission, one from the paternally inherited allele and the other from the maternally allele. As a consequence of these randomizations, genotypes are not expected to be associated with known or unknown confounders for any outcome of interest, except those lying on the causal pathway between the genotype and the outcome. This allows analyzing the genotype-risks factor association (in this case, the genotype-the plasma total homocysteine) and the genotype-outcome association (in this case, the genotype-schizophrenia) in an unconfounded manner [13]. By combining the results of the genotype-risk factor association and the genotype-outcome association, one can get an estimate of the risk factor-outcome association (in this case, the plasma total homocysteine-schizophrenia). In addition, genetic variants are equivalent to lifetime differences in risk factor (in this case, the plasma total homocysteine), and indicate the long term effects of risk factor on disease (in this case, schizophrenia). They therefore generate more realistic estimates of causal effects between risk factor and disease [5]. The Mendelian randomization approach has similar properties to the analysis of the intention to treat in randomized controlled trials [5, 17], and this approach has provided new insights into the pathology of several diseases, such as cardiovascular disease, diabetes, and Parkinson disease [18–26].

In this study, we evaluated a causal relationship between plasma total homocysteine and schizophrenia by conducting a Mendelian randomization analysis based on the *MTHFR* C677T polymorphism as an instrumental variable.

## Methods

### The estimate for gene-schizophrenia association

The risk estimate for the gene-schizophrenia association of the T allele of the *MTHFR* C677T polymorphism (OR _scz/per T-allele_) was evaluated by conducting a meta-analysis of case–control studies with the random-effect model by ‘metafor’, an R package. Eligible studies were identified using SZGene [27] and the PubMed search engine with the terms “Methylenetetrahydrofolate reductase” or “*MTHFR*” or “schizophrenia”. We also conducted an additional manual search of reference lists and review articles. Studies meeting the following criteria were included for meta-analysis: (1) performed a case–control study (i.e., schizophrenia versus control), (2) provided data on genotype frequencies, and (3) was published in the English language. The two reviewers (Kinoshita M and Nishi A) selected the articles independently according to the above inclusion criteria, and then discussed the articles until they reached a consensus on every study used for the meta-analysis. Heterogeneity was assessed using the I^2^ statistic, and publication bias was assessed using funnel plots and a regression test [28]. OR and 95 % confidence intervals (CI) were calculated by ‘metafor’, an R package. Finally, we used 36 case–control studies from 32 papers for a total of 11,042 patients with schizophrenia and 14,557 control subjects [4, 29–59] based on a Preferred Reporting Items for Systematic Review and Meta-Analyses (PRISMA) flowchart [60] (Additional file 1: Figure S1).

### The estimate for gene-plasma total homocysteine association

We used a pooled estimate of per-T allele standardized β coefficient (0.158) of the effect of the *MTHFR* C677T polymorphism on the natural log-transformed plasma total homocysteine levels (beta _hcy/per T-allele_) from a recent meta-analysis of genome-wide association studies comprising 44,147 individuals [2].

### Mendelian randomization analysis

We calculated a Mendelian randomization estimate of the effect of the plasma total homocysteine levels on the risk of schizophrenia (OR _scz/hcy_) as log OR _scz/hcy_ = (log OR _scz/per T-allele_)/ beta _hcy/per T-allele_, as in previous studies [4, 21]. Log OR _scz/hcy_ is the (log) increase of schizophrenia risk by 1-standard deviation (SD) increase in the natural log-transformed plasma total homocysteine (plasma total homocysteine-schizophrenia association). Log OR _scz/per T-allele_ is the (log) increase in schizophrenia risk per T allele of the *MTHFR* C677T polymorphism (gene-schizophrenia association). Beta _hcy/per T-allele_ is the number of SD differences in the natural log-transformed plasma total homocysteine levels per the T allele (SD/allele) (gene-plasma total homocysteine association).

## Results

**Fig. 1:**
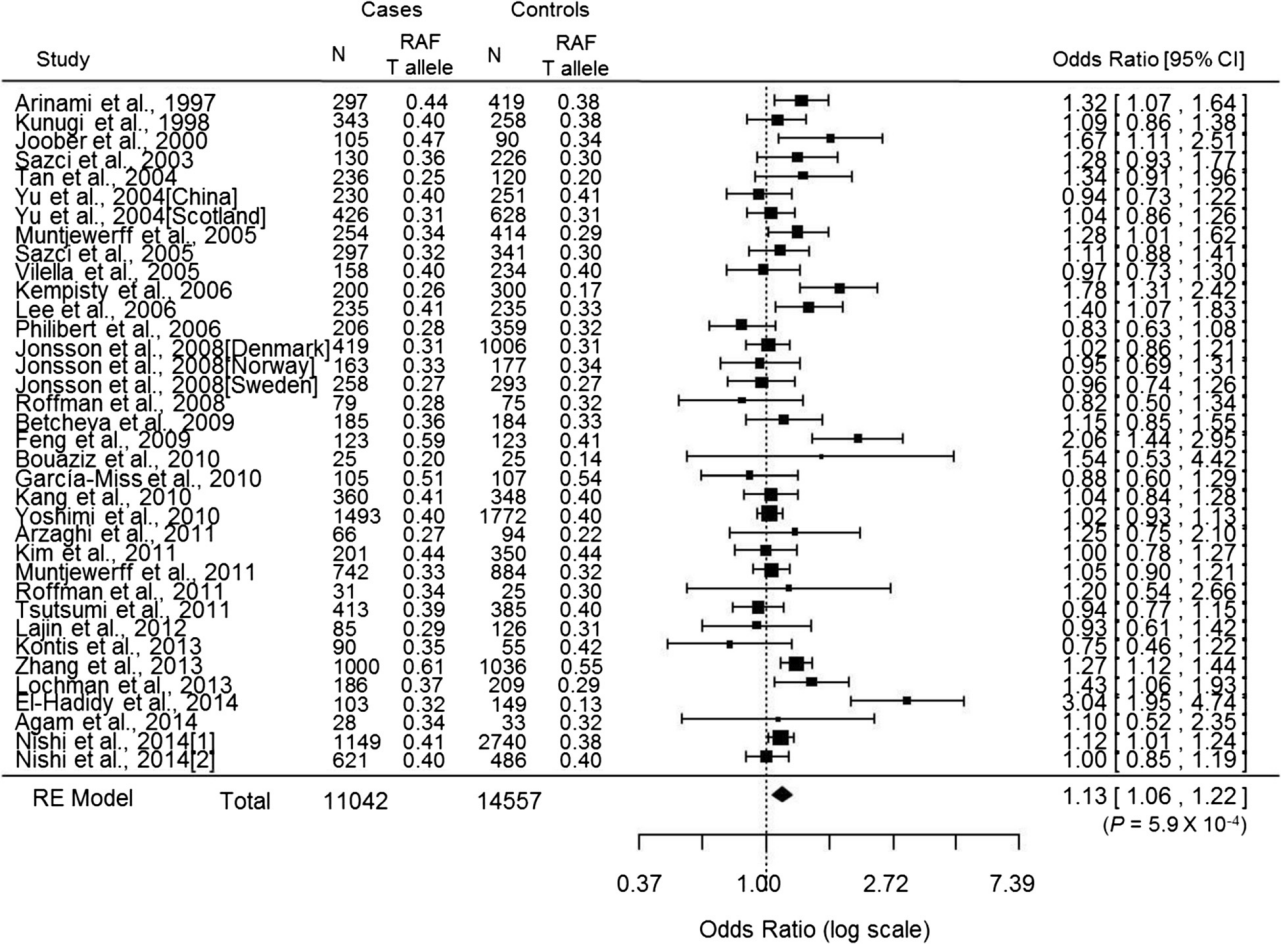
A meta-analysis of genetic association studies between the *MTHFR* C677T polymorphism and schizophrenia. Thirty six case–control studies were used for the meta-analysis (N = 25,599). The pooled OR per T allele of the *MTHFR* C677T polymorphism on schizophrenia risk was 1.13 (95 % CI = 1.06–1.22; *p* = 5.9 x 10^−4^) in the random-effects model with significant heterogeneity among studies (I^2^ = 66.1 %; *p* < 0.05)

**Fig. 2:**
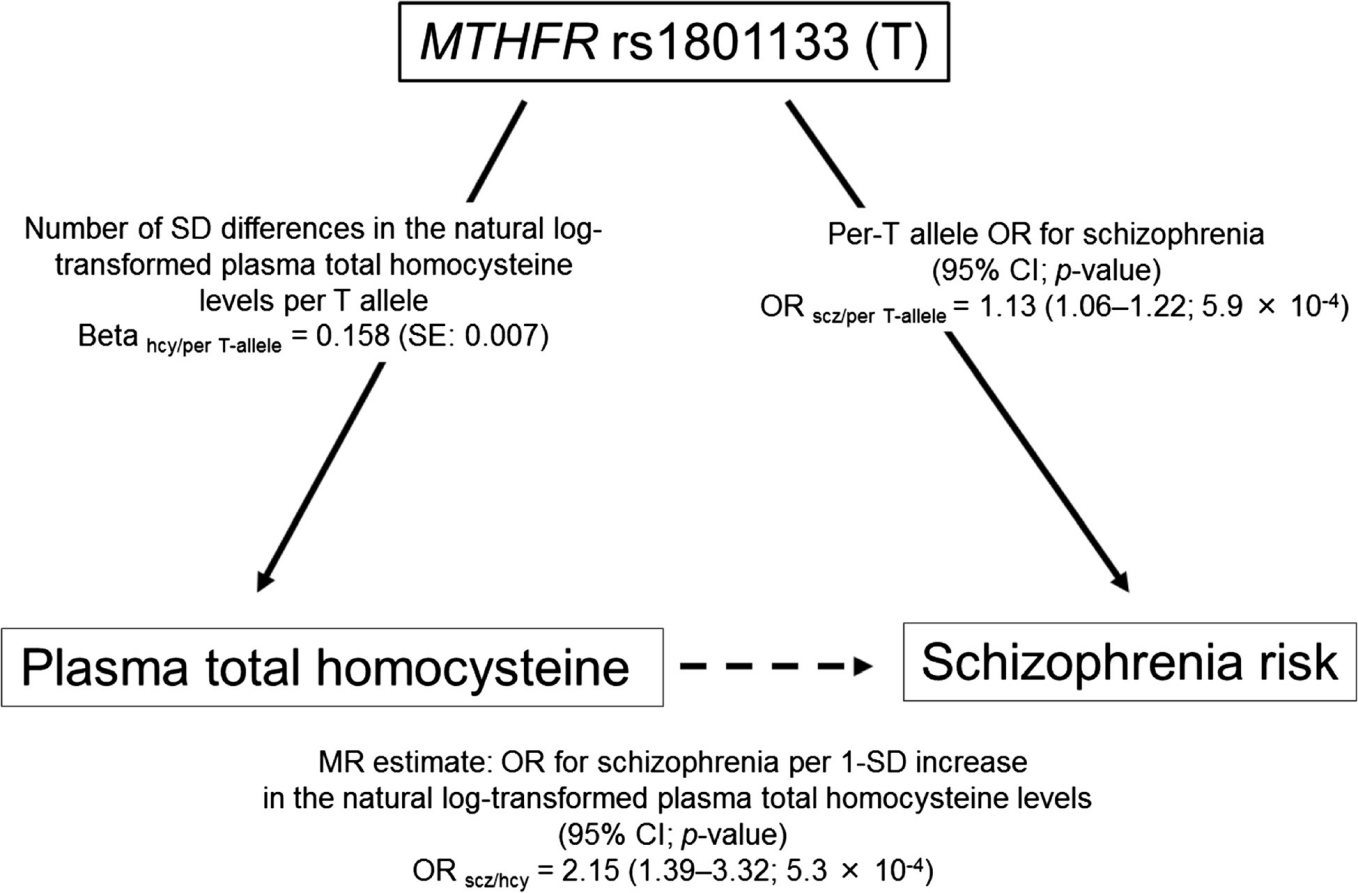
Graphical representation of the Mendelian randomization approach. The risk estimate for the gene-schizophrenia association of the T allele of the *MTHFR* C677T polymorphism was obtained from the present meta-analysis of previous genetic association studies that cover a total of 25,599 individuals (11,042 cases and 14,557 controls). To establish the gene-homocysteine association, we took a pooled estimate of the effect of the *MTHFR* C677T polymorphism on the plasma total homocysteine levels from a recent meta-analysis of previous genome-wide association studies, including 44,147 individuals (van Meurs et al. [2]). From these two estimates, we calculated a Mendelian randomization estimate of the effect of the plasma total homocysteine levels on the risk of schizophrenia. This estimate represented the OR for schizophrenia risk per 1-SD increase in the log-transformed plasma total homocysteine levels

The pooled OR _scz/per T-allele_ was 1.13 (95 % CI = 1.06–1.22; *p* = 5.9 x 10^−4^ in the random-effects model) with significant heterogeneity (I^2^ = 66.1 %; *p* < 0.05; Fig. 1). The funnel plot analysis indicated no evidence of publication bias in this meta-analysis (*p* > 0.05). By combining two pooled estimates, OR _scz/per T-allele_ from a meta-analysis of 36 case–control studies and beta _hcy/per T-allele_ from a meta-analysis of genome-wide association studies by van Meurs and colleagues [2], we found a significant effect of the plasma total homocysteine on schizophrenia risk in the Mendelian randomization analysis, representing an OR _scz/hcy_ of 2.15 (95 % CI = 1.39–3.32; *p* = 5.3 x 10^−4^) for schizophrenia per 1-SD increase in the natural log-transformed plasma total homocysteine levels (Fig. 2).

## Discussion

By conducting a Mendelian randomization approach, we demonstrated that increased plasma total homocysteine concentration levels may be causally associated with an increased risk of developing schizophrenia. Our finding is consistent with the result of our recent paper that used a Japanese cohort [4]. Our finding is also supported by a longitudinal study in which elevated maternal levels of homocysteine levels during the third trimester were found to increase the risk of schizophrenia in the offspring [61]. Furthermore, the benefits of homocysteine-reducing strategies in schizophrenia have been reported in previous studies with randomized, double-blind, and placebo-controlled designs. Levine et al. [62] reported an improvement in the clinical symptoms of the patients with schizophrenia and hyper-homocysteinemia (over 15umol/L) who were treated with folate, vitamin B12, and pyridoxine. Roffman et al. [63] reported an improvement in the negative symptoms of patients with schizophrenia who were treated with folate and vitamin B12 when several functional genetic variants were taken into account.

Hyper-homocysteine has also been observed in cardiovascular disease [64], and yet randomized trials have failed to demonstrate benefit of homocysteine-lowering intervention on cardiovascular outcomes [18, 65]. The discordant results from observational studies and randomized trials might be caused by the limited period of the randomized trials, the effects of aspirin and other antiplatelet drugs [66], confounding factors, or reverse causation. Several papers had not found evidence in support of a causal association between homocysteine and cardiovascular disease [2, 18].

There are some limitations to the present Mendelian randomization analysis. One is the number of genetic variants. We used one polymorphism as the instrumental variable that affects the plasma total homocysteine concentrations. However, a recent meta-analysis of genome wide association studies of the plasma total homocysteine has identified several genetic variants [2]. Further replication studies will be needed using multiple genetic variants related to the plasma total homocysteine levels, because using multiple instruments increases the precision of the instrumental variable estimates [14]. The second is a reintroduced confounding through pleiotropy [12, 14, 15]. Pleiotropy is defined as the phenomenon in which a single locus affects two or more distinct phenotypic traits [67]. The *MTHFR* C677T polymorphism may directly influence more than one post-transcriptional process. The third is population stratification [12, 14, 15]. When we estimated both gene-schizophrenia association and gene-plasma total homocysteine association, we used a cohort that was composed of a mixed population. The fourth is a developmental compensation [12, 15]. During development, compensatory process may be generated that counter the phenotypic perturbation consequent on the genetic variant utilized as an instrument [15].

## Conclusions

In summary, we provided evidence of a causal relationship between the plasma total homocysteine and schizophrenia by conducting a Mendelian randomization approach. However, our findings have to be interpreted with caution because of the limitations, such as pleiotropy and population stratification.

### Funding body agreements and policies

This work was supported in part by Japan Science and Technology Agency, CREST and a Grant-in-Aid for Scientific Research from the Japanese Ministry of Education, Culture, Sports, Science and Technology (grant number 24791216 and 26860931), SENSHIN Medical Research Foundation, and the Research Group for Schizophrenia.

## Additional file

Additional file 1: Figure S1. A flowchart of study selection using a PRISMA checklist. The number of records identified through database searching was 234. After adjusting for duplicates, 113 studies were remained. By checking the titles, we excluded studies published in languages other than English (8 studies), review and case reports (13 studies), and studies using animal samples (4 studies). By reviewing abstracts, we excluded studies which were not case–control studies (34 studies), a study using cases without schizophrenia (1 study), and a study examining a different single-nucleotide polymorphisms (1 study). After these steps, 52 studies were remained. Of the 52 studies that were subjected to full-text inspection, we further excluded studies which did not contain original data (8 studies), studies which did not compare cases with controls (5 studies), a study which did not contain the details of samples (1 study), a study using cases without schizophrenia (1 study), and studies which were suspected overlapping of samples (5 studies). Finally, 32 papers, including 36 case–control studies, were remained.
