# Supplementary material for: Evaluation of an association between plasma total homocysteine and schizophrenia by a Mendelian randomization analysis
Source: BMC Med Genet. 2015 Jul 26;16:54. doi: 10.1186/s12881-015-0197-7 (PMC4557634; doi:10.1186/s12881-015-0197-7)
Supplement: Additional file 1: Figure S1. — A flowchart of study selection using a PRISMA checklist. The number of records identified through database searching was 234. After adjusting for duplicates, 113 studies were remained. By checking the titles, we excluded studies published in languages other than English (8 studies), review and case reports (13 studies), and studies using animal samples (4 studies). By reviewing abstracts, we excluded studies which were not case–control studies (34 studies), a study using cases without schizophrenia (1 study), and a study examining a different single-nucleotide polymorphisms (1 study). After these steps, 52 studies were remained. Of the 52 studies that were subjected to full-text inspection, we further excluded studies which did not contain original data (8 studies), studies which did not compare cases with controls (5 studies), a study which did not contain the details of samples (1 study), a study using cases without schizophrenia (1 study), and studies which were suspected overlapping of samples (5 studies). Finally, 32 papers, including 36 case–control studies, were remained. [file 12881_2015_197_MOESM1_ESM.doc]

**Additional file 1: Figure S1. A flowchart of study selection using a PRISMA checklist.**

**Screening**

**Included**

**Eligibility**

**Identification**

Records identified through database searching
(n = 234 )

Additional records identified through other sources
(n = 0 )

Records after duplicates removed
(n = 113 )

Records underwent title and abstract screening
(n = 113 )

Records excluded
(n = 61 )

Full-text articles assessed for eligibility
(n = 52 )

20 articles excluded after full text review:

-Without original data (n = 8)

-No case-control (n = 5)

-Without details about sample and results (n =1)

-No target outcomes (n = 1)

-Overlap study (n = 5)

Studies included in quantitative synthesis (meta-analysis)
(n = 32 )
